# A case report of splenic malignancy originating from the liver

**DOI:** 10.3389/fonc.2026.1863458

**Published:** 2026-06-04

**Authors:** Lei Shen, Yechuan Xu, Jun Li, Zixuan Yang

**Affiliations:** 1 Department of General Surgery, The Third People's Hospital of Hefei, Hefei Third Clinical College of Anhui Medical University, Hefei, Anhui, China; 2 Department of General Surgery, The First Affiliated Hospital of Anhui Medical University, Hefei, China

**Keywords:** case report, hepatocellular carcinoma, splenic metastasis, splenic tumor, treatment

## Abstract

**Introduction:**

Splenic tumors are infrequently encountered in clinical practice, with a metastatic tumor incidence estimated to be approximately 2%-4% of all splenic tumors. Metastatic tumors originating from the liver are even more uncommon. Given the scarcity of reported cases and limited literature on splenic tumors, particularly in recent years, clinicians face significant challenges in effectively managing this disease.

**Case presentation:**

In this report, we present a case of a malignant splenic tumor originating from the liver. The patient, a 58-year-old male, was admitted to our hospital over a week after the identification of a splenic mass. Notably, the patient had previously undergone liver cancer surgery at our institution two years prior. The current diagnosis is recurrent liver cancer, secondary malignant tumor of the spleen, and gallbladder stones. Based on the patient’s clinical presentation, physical examination findings, and additional diagnostic tests, we performed a surgical procedure known as “ left hepatic lateral lobectomy, splenectomy and cholecystectomy”. The postoperative pathological analysis confirmed the presence of recurrent hepatocellular carcinoma with splenic metastasis. Up to the last follow-up, the patient was in stable general condition without obvious discomfort and remained alive.

**Conclusion:**

Combining domestic and international literature, we now report a case of splenic malignancy of hepatic origin. In this case report, we aim to present its differential diagnosis and treatment options for clinicians’ reference.

## Introduction

The spleen tumor is a rare clinical condition that is divided into primary and secondary tumors. Primary benign tumors include hemangiomas, lymphomas, fibromas, lipomas, and malignant tumors (1, 2). The incidence of these primary tumors is about 0.03% of the whole body tumors. Malignant tumors are divided into two categories: primary and secondary. Primary malignant tumors are all sarcomas, such as fibrosarcoma, lymphosarcoma, hemangiosarcoma, and reticulocyte sarcoma. Among them, lymphosarcoma accounts for 20%. Angiosarcoma may occur in the spleen or it may be metastatic. Metastatic tumors of the spleen occur in approximately 2%-4% of all splenic tumors, with the undifferentiated type being the most common. The primary sites are mostly lung, stomach, pancreas, and colon, followed by choriocapillaris epithelial carcinoma, malignant melanoma, and breast cancer. Liver origin is extremely rare.

Despite the continuous improvement of medical technology in recent years, its incidence has not increased, and it is even less reported in the literature. Therefore, combining domestic and international literature, we are reporting a case of secondary malignancy of the spleen of hepatic origin. In this case report, we aim to present its differential diagnosis and treatment options for clinicians’ reference.

## Case presentation

**Figure 1:**
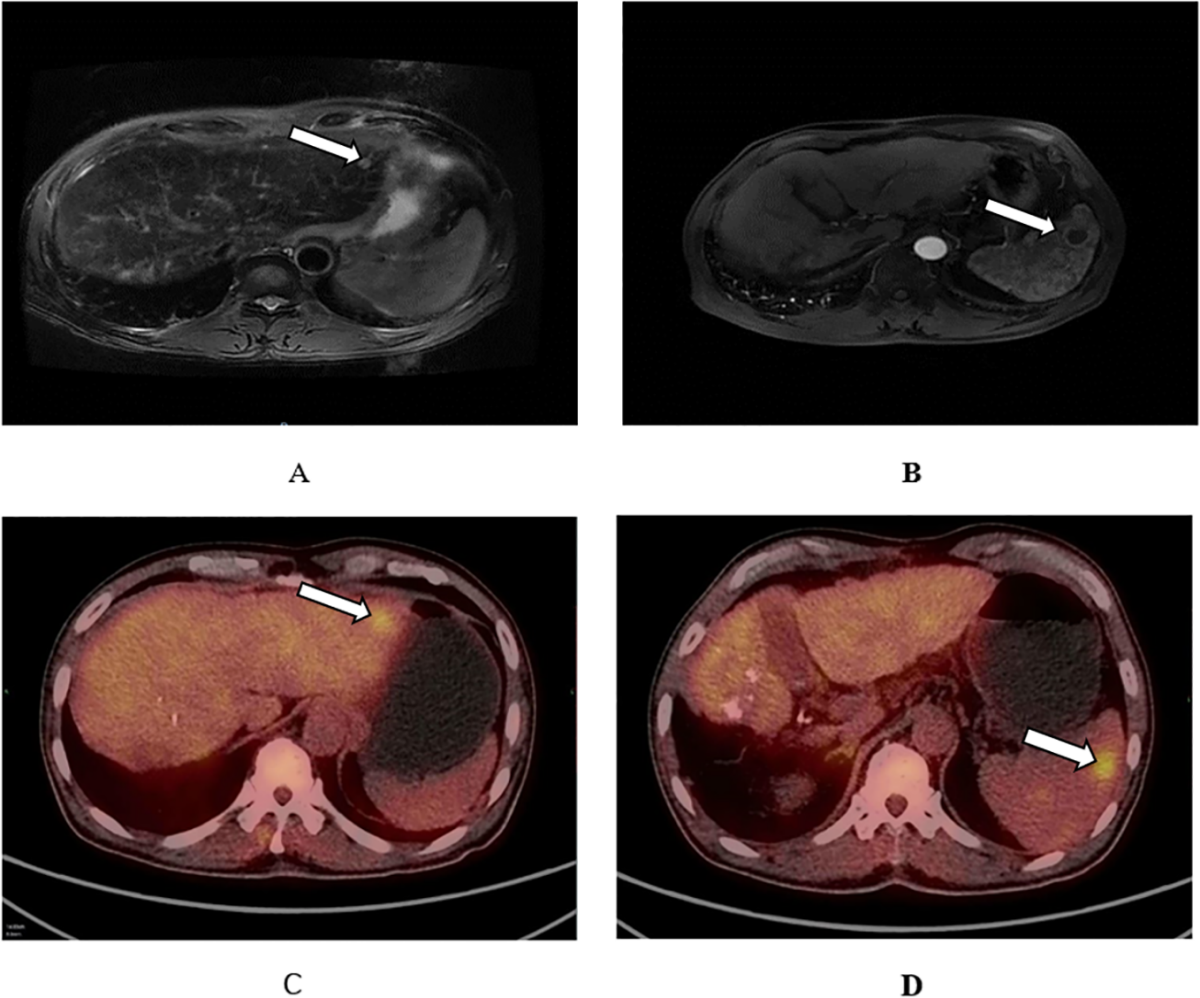
MRI and PET-CT images of metastatic tumors in the spleen. Panel **(A)** shows MRI. Plain T2-weighted images (T2WI). Panel **(B)** shows enhanced scan images. Panel **(C)** shows FDG hypermetabolic images of metastatic tumors in the left outer lobe of the liver. Panel **(D)** shows. The FDG hypermetabolic image of a splenic metastatic tumor. The location of the lesion has been marked with an arrow.

**Figure 2:**
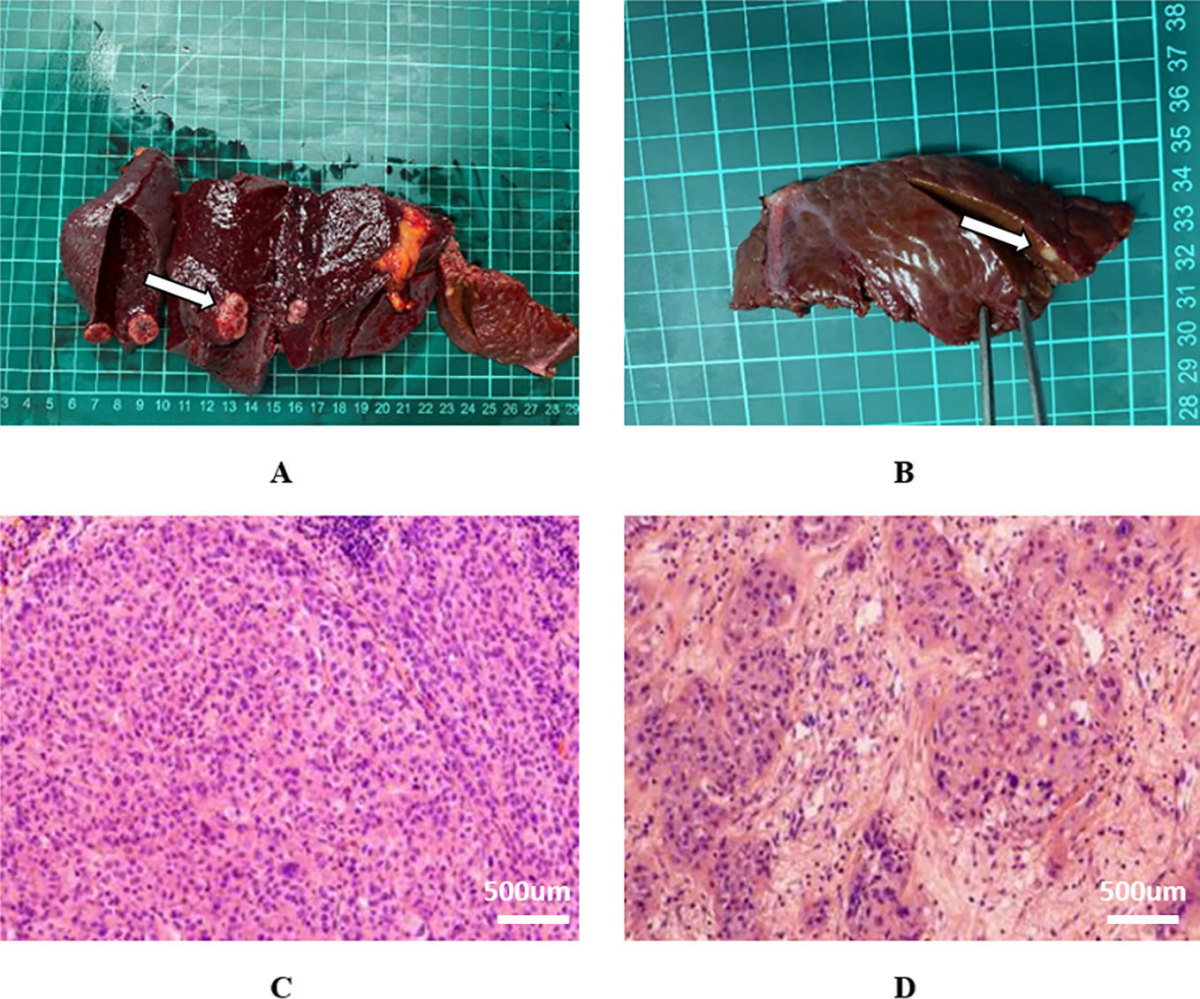
Postoperative specimen and postoperative pathological pictures of the patient. Panel **(A)** shows metastatic tumor in the spleen; Panel **(B)** shows the left outer lobe of the liver with one metastatic tumor visible. Panels **(C, D)** show the postoperative pathological pictures of the patient (HE staining, ×40). The location of the lesion has been marked with an arrow.

The patient, a 58-year-old male, was admitted with a splenic occupancy. The patient underwent right posterior lobe resection of the liver in our hospital two years ago after an MDT discussion following the diagnosis of liver occupancy, and postoperative pathology showed hepatocellular carcinoma. He was followed up regularly in the outpatient clinic later. Now the patient’s examination revealed abnormal signals in the spleen, and metastasis was considered, so he was consulted at our hospital. The patient’s history was unremarkable and there were no significant positive signs on physical examination of the abdomen. After admission to the hospital to complete the relevant blood and imaging tests, our blood tests showed: white blood cell count 4.07×10^9^/L, red blood cell count 4.56×10¹²/L, hemoglobin 149 g/L, platelet count 46×10^9^/L; serum albumin 49.7 g/L, alanine transaminase 26 U/L, aspartate transaminase 34 U/L; alpha-fetoprotein 1.92 ng/mL, and cancer antigen 125–12 ng/mL. Our imaging showed: PET-CT: recurrent hepatocellular carcinoma with metastasis to the left outer lobe of the liver; with liver tumor size approximately 0.6×0.5 cm. The size of the spleen tumor is approximately 1.5 ×1.2cm; multiple metastases to the spleen; gallbladder stones with chronic cholecystitis. No obvious metastatic lesions were observed in other organs. Enhanced MRI showed: postoperative changes in the liver and abnormal signals in the spleen (**Figures 1**, **2**). Combining the patient’s clinical manifestations, physical examination, and ancillary tests, we performed “ left hepatic lateral lobectomy, splenectomy and cholecystectomy. The operation lasted for approximately 3 hours. The surgical bleeding was 200 milliliters. No blood transfusion was required. A drainage tube was placed in the splenic fossa. No enlarged lymph nodes were found during the operation. The patient recovered well after the surgery and no complications occurred. The postoperative pathology was as follows: The liver lesion was hepatocellular carcinoma (moderate-low differentiation), consistent with recurrence of liver cancer. The tumor was closely adjacent to the liver resection margin (<0.1cm) and no satellite nodules were observed. The spleen lesion was a malignant tumor, approximately 1.5cm x 1.2cm x 0.9cm in size. Its histological image was similar to the intrahepatic nodule, with poor tumor differentiation and the tumor invading the spleen parenchyma. No cancer metastasis was found in the splenic hilar fat and the three lymph nodes (0/3).The postoperative pathological diagnosis was recurrent hepatocellular carcinoma with splenic metastasis. The immunohistochemistry of the patient was: Hepatocyte (partial +), GPC3 (+), AFP (weak +), CK19 (bile duct +), CK7 (bile duct +), CK8 (+), CD34 (vascular endothelial +), Ki-67 (about 40%, +) (**Table 1**).

**Table 1:** Baseline oncologic and hepatologic data.

status of viral hepatitis	Chronic viral hepatitis B.
liver function score	Child-Pugh grade A
tumor stage	T_1_N_0_M_1_
tumor markers	alpha-fetoprotein 1.92 ng/mL
Clinical presentation	Upper abdominal pain; ECOG PS1
comorbidities	Chronic viral hepatitis B, Hypertension, gallbladder stones, malignant liver tumor
previous treatments	After liver resection, long-term use of antiviral drugs
pathological features of the original HCC.	Poorly differentiated liver cancer; Negative margins; Single tumor; Microvascular infiltration; The envelope is intact. No lymph node metastasis

The patient was discharged from the hospital 15 days after surgery and was in good general condition with normal laboratory test results. We recommend regular follow-ups in the outpatient clinic.

The last outpatient follow-up was in November 2024 and the patient had normal tumor markers **Table 2**. Ultrasound and MRI showed no signs of recurrence. During the follow-up period, the patient was compliant and able to attend regular outpatient visits without adverse events. This research has been approved by the ethics committee, and the approval number is 2026LLWL021.

**Table 2:** Treatment timeline.

March 16, 2020	The first partial hepatectomy was performed
April 21, 2020	Hepatic artery embolization chemotherapy was performed (TACE)
June 01, 2020	Hepatic artery embolization chemotherapy was performed (TACE)
June 19, 2020	Immunotherapy, Sintilimab (anti-PD-1 monoclonal antibody) was administered intravenously at a dose of 200 mg every 3 weeks as postoperative adjuvant therapy
September 28, 2020	Hepatic artery embolization chemotherapy was performed(TACE)
November 14, 2021	Immunotherapy
January 19, 2022	Hepatic artery embolization chemotherapy was performed(TACE)
March 28, 2022	Immunotherapy
June14, 2022	left hepatic lateral lobectomy, splenectomy and cholecystectomy
June29, 2022	Discharge from hospital
August 19, 2022	Hepatic artery embolization chemotherapy was performed (TACE)
November 28, 2024	Latest followed-up

## Discussion

Metastatic tumors of the spleen are a rare clinical condition with a low incidence despite abundant blood flow to the spleen. We found that malignant tumors that commonly metastasize to the spleen include melanoma and tumors originating from the breast, lung, ovary, colon, stomach, and pancreas, while malignant tumors originating from the liver are extremely rare in combination with domestic and international literature (3). To address the reasons for this, it has been reported in the literature that firstly, due to the characteristics of the splenic vascular anatomy, the splenic artery divides from the abdominal aorta at a large angle, so that cancer emboli do not easily enter; secondly, the spleen can contract rhythmically; thirdly, the spleen has no input lymphatics; and finally, the spleen has abundant lymphoid tissue, which is immunologically powerful (4–6). The coexistence of these factors results in a low incidence of metastatic tumors in the spleen.

The clinical manifestations of metastatic tumors of the spleen are atypical, and small lesions may have no clinical symptoms. Huge ones show splenomegaly and discomfort and pain in the left upper abdomen, or nausea, vomiting, and abdominal distension due to gastrointestinal pressure. Malignant cases show rapid enlargement of the spleen with a nodular surface. In addition to left upper abdominal pain and gastrointestinal pressure, there were also signs of wasting, anemia, fever, cachexia, and mild jaundice. We found that in imaging, metastatic tumors in the spleen mostly appear as hypoechoic in ultrasound, but it is impossible to differentially diagnose benign and malignant tumors in the spleen, and CT examination shows one of the most diagnostic methods at present, and in enhanced CT examination, metastatic tumors in the spleen appear as single or multiple hypodense foci in the spleen with unclear borders; there can be mild enhancement after enhancement. MRI is more sensitive than CT examination, and its performance is most clear in the portal phase (7, 8). The incidence of calcification in metastatic tumors of the spleen is extremely low, except for specific pathological types, such as primary tumor splenic mucinous adenocarcinoma. In addition, we also need to conduct a differential diagnosis for the lesions of the spleen, such as primary splenic angiosarcoma, lymphoma, littoral cell angioma, abscess/granulomatous diseases, and benign spleen lesions. Splenic angiosarcoma progresses rapidly and is prone to early lung metastasis. The most common manifestation of splenic lymphoma is the enlargement of the entire lymphatic system throughout the body. littoral cell angioma usually do not have invasive properties; abscess/granulomatous lesions usually have a history of infection, systemic infection symptoms, and abnormal laboratory pathogen findings. Therefore, it is especially important to combine many factors and how to make an accurate diagnosis. This patient had a history of liver cancer surgery. During this admission, liver and spleen lesions were discovered. In order to determine the nature of the lesions in the spleen and liver, a biopsy should be performed. However, considering that the spleen and liver have abundant blood supply, the biopsy may result in significant bleeding. Based on the imaging examination results, we have a high suspicion that the spleen has developed malignancy. Therefore, we decided to abandon the preoperative biopsy. After evaluating the resectable tumor and seeking the family’s consent, we performed “ left hepatic lateral lobectomy, splenectomy and cholecystectomy “, and the postoperative pathology was: recurrent hepatocellular carcinoma with splenic metastasis.

In conclusion, metastatic tumors of the spleen are extremely rare and there are many different treatments available. These include conservative medical treatment and surgical treatment. For resectable primary malignant tumors of the spleen, surgery should be the main treatment. The principle of surgery for primary malignant tumors of the spleen is complete splenectomy, do not rupture the tumor during surgery to avoid implantation. If the lymph nodes of the splenic hilum are involved, lymph node dissection should be performed; if the tumor invades the adjacent organs, joint resection of the adjacent organs should be pursued. For metastatic splenic tumors, we need to carefully evaluate the primary focus and the presence of other distant metastases preoperatively and strive for resection if there is an isolated lesion in the spleen. Since splenic malignant tumors are prone to hematogenous metastasis, comprehensive therapeutic measures such as radiotherapy and immunotherapy should be actively taken after surgery to improve the survival of patients. The patient in this case was selected for surgical treatment after preoperative discussions due to a secondary malignancy of the spleen of hepatic origin. Postoperatively, we used an individualized treatment plan for the patient’s condition. The protocol is transhepatic arterial chemoembolization + immunotherapy. The patient has no recurrence in postoperative follow-up to date, and the patient is generally doing well. Therefore, for isolated and resectable metastatic tumors of the spleen, if the patient has good liver function and overall health, and no other distant metastases are present, we may consider adopting surgical treatment combined with postoperative chemotherapy and immunotherapy to improve the patient’s survival rate.

## Patient’s statement

We contacted the patient in 28 November 2024 and asked him for his views and insights on our treatment. The patient said he was very satisfied with the treatment his doctors had chosen for him throughout the treatment process. He said he suffered from the ravages of the disease and was in great pain. Thanks to the correct treatment by doctors, he was able to resume a normal life. He also said that he agreed to report his disease so that more patients could be treated.

## Data Availability

The original contributions presented in the study are included in the article/supplementary material. Further inquiries can be directed to the corresponding author.
